# High-throughput 3D phenotypic screening identifies repurposed MEK inhibitors as drivers of chondrogenesis for cartilage regeneration

**DOI:** 10.3389/fbioe.2026.1748443

**Published:** 2026-02-23

**Authors:** Hadi Hajiali, Justyna Cholewa-Waclaw, Jacob Ballard, Kerime Ebrar Okur, Richard Elliott, Neil O. Carragher, Alicia J. El Haj

**Affiliations:** 1 Healthcare Technologies Institute, Institute of Translational Medicine, School of Chemical Engineering, University of Birmingham, NIHR Birmingham Biomedical Research Centre, Birmingham, United Kingdom; 2 Institute for Regeneration and Repair, University of Edinburgh, Edinburgh, United Kingdom; 3 Cancer Research UK Scotland Centre, Institute of Genetics and Cancer, University of Edinburgh, Edinburgh, United Kingdom

**Keywords:** 3D cartilage regeneration, chondrogenesis, drug repurposing, high-throughput drug screening, MEK inhibitor, trametinib

## Abstract

**Background and purpose:**

Chondrogenesis is essential for cartilage repair and regeneration, particularly in treating osteoarthritis and cartilage injuries. While conventional therapies rely heavily on growth factors, recent interest has turned toward drug repurposing strategies involving small-molecule inhibitors. This study aims to evaluate the chondrogenic potential of selected bioactive compounds, with a particular focus on Trametinib, a MEK inhibitor.

**Experimental approach:**

A library of 55 bioactive compounds was screened using high-content imaging and a 3D hydrogel model that mimics the native cartilage microenvironment. Cellular morphology, migration, and cytoskeletal organization were assessed to identify chondrogenic phenotypes. Trametinib, along with Panobinostat, SAHA, and Brefeldin A, was further evaluated via dose-response analyses and molecular assays to determine their impact on chondrogenic differentiation.

**Key Results:**

Trametinib was identified as a potent modulator of chondrogenesis-related cellular phenotypes. It significantly altered cell morphology, promoted a chondrogenic-like shape, and enhanced cell migration. Changes in actin organization were quantified using SER-Spot and SER-Ridge metrics, showing patterns consistent with chondrogenic differentiation. Molecular analysis revealed upregulation of Collagen II and aggrecan, key markers of cartilage formation.

**Conclusion and implications:**

These findings support the potential of MEK inhibitors like Trametinib, and other selected bioactive compounds, as promising agents for cartilage regeneration. Their repurposing could offer innovative therapeutic strategies for treating cartilage-related disorders, including osteoarthritis.

## 1 Introduction

Chondrogenesis, the process by which cartilage is formed, plays a crucial role in maintaining healthy cartilage tissue and is essential for cartilage repair, regeneration, and the treatment of cartilage-related diseases (Imere et al., 2024; Foster et al., 2022). As a result, finding effective therapeutic strategies that can stimulate chondrogenic differentiation is of great interest for regenerative medicine, particularly for conditions like osteoarthritis (OA) and cartilage injuries (Foster et al., 2015). Traditional approaches to promoting chondrogenesis include the use of chondrogenic media, which typically contains growth factors such as transforming growth factor-beta (TGF-β), insulin-like growth factor (IGF), and bone morphogenetic proteins (BMPs) (Du et al., 2023). While these factors are well-established in promoting chondrocyte differentiation, there is a growing need for alternative or complementary therapies that can enhance chondrogenesis without relying solely on growth factor supplementation.

In addition to growth factor therapy, clinical cartilage-based therapies have advanced, offering various approaches to repair and regenerate cartilage in OA and other degenerative conditions. Mesenchymal stem cell (MSC) therapy is one such clinical approach; MSCs derived from sources such as bone marrow or adipose tissue can differentiate into chondrocytes, potentially enhancing cartilage repair and reducing joint inflammation (Lee and Wang, 2017; Carneiro et al., 2023). Another widely studied therapy, platelet-rich plasma (PRP) injections, utilizes the patient’s own blood to produce a plasma rich in growth factors that can promote tissue healing and reduce inflammation in cartilage (Yurtbay et al., 2022; Liang et al., 2022). Autologous chondrocyte implantation (ACI) and matrix-induced autologous chondrocyte implantation (MACI) involve harvesting healthy chondrocytes from the patient, expanding them *in vitro*, and reimplanting them into cartilage lesions. These procedures are widely used for focal cartilage defects and have shown promising outcomes in terms of cartilage regeneration (Migliorini et al., 2021; Migliorini et al., 2022). Despite these advancements, the complex structure and limited vascularity of cartilage still present challenges for complete cartilage regeneration, prompting the search for additional strategies that can improve therapeutic outcomes (Carneiro et al., 2023; Yurtbay et al., 2022).

Recent advances in drug repurposing have opened new avenues for identifying compounds that modulate chondrogenic pathways. Small-molecule inhibitors, originally developed for cancer therapies, have shown promise in inducing chondrogenic differentiation and enhancing tissue regeneration. Cancer drugs, particularly methotrexate and Janus kinase (JAK) inhibitors, have been repurposed for cartilage-related therapies, especially in autoimmune diseases like rheumatoid arthritis (RA) that lead to joint damage (Fang et al., 2022; Angelini et al., 2020). Methotrexate, a common chemotherapeutic agent, is used at lower doses in RA to suppress immune cell activity, reducing inflammation and subsequent cartilage degradation (Fang et al., 2022). JAK inhibitors such as tofacitinib and baricitinib, initially developed for malignancies due to their role in immune signalling, are now used to manage RA by inhibiting the JAK pathway, effectively reducing inflammation and slowing disease progression in cartilage tissues (Angelini et al., 2020; Kiełbowski et al., 2024). Furthermore, tumour necrosis factor (TNF) inhibitors are now integral to RA treatment (Ma and Xu, 2013; George et al., 2020). By blocking tumour necrosis factor-alpha (TNF-α), a key pro-inflammatory cytokine, these inhibitors help prevent cartilage damage and reduce joint inflammation in RA. The repurposing of cancer drugs for cartilage treatment underscores the potential of targeting specific signalling pathways to promote chondrogenesis and control cartilage inflammation, however, these drugs are predominantly focused on modulating inflammation and the direct effects on chondrogenic cell differentiation of behaviour are less well understood.

Recent advances in high throughput phenotypic screening technologies such as automated high content imaging are impacting upon all stages of modern drug discovery and identification of drug repurposing opportunities (Way et al., 2023). In this high content screening study, we screened 55 bioactive compounds across a range of concentrations to assess their effects on key cellular parameters such as cell morphology, protein expression, migration, and proliferation, as well as their influence on cytoskeletal organization, for their ability to induce chondrogenesis *in vitro*. Trametinib, a MEK inhibitor approved for the treatment of melanoma (Attwood et al., 2021; U.S.F. and D. Administration, 2018), has emerged as a potential candidate for modulating signaling pathways involved in cell differentiation and proliferation (Kim M.-Y. et al., 2023). The mitogen-activated protein kinase (MAPK) signaling pathway, particularly through mitogen-activated protein kinase (MEK) and extracellular signal-regulated kinase (ERK), plays a crucial role in regulating various physiological processes, including cell proliferation, differentiation, survival, and apoptosis (Li et al., 2007; Chambard et al., 2007). In the context of cartilage development, activation of the MEK/ERK pathway inhibits chondrocyte differentiation, while its inhibition has been shown to promote the differentiation of mesenchymal stem cells (MSCs) into chondrocytes and enhances chondrogenesis (Wang et al., 2018; Stanton et al., 2003; Bobick and Kulyk, 2004).

In this study, we adapted a recently developed novel 3D culture model using GelMA hydrogel scaffolds (Imere et al., 2024), which more closely mimic the extracellular environment of cartilage tissue compared to traditional 2D cultures. It enables us to monitor multiple parameters relevant to chondrogenesis such as migration, differentiation and proliferation in the same screen. The use of engineered hydrogel systems enables precise control over both biochemical and biomechanical cues, which are increasingly recognized as critical regulators of chondrogenesis and cartilage regeneration. Recent studies have demonstrated that spatially graded and bioinspired scaffolds can more effectively recapitulate the complex structure of osteochondral tissues and promote region-specific cellular responses (Peng et al., 2023; Wen et al., 2024). Building on these advances, our 3D GelMA platform provides a mechanically tunable and biologically relevant environment that enables high-content phenotypic screening of small-molecule libraries. This approach allows simultaneous assessment of key chondrogenesis-related parameters, including cell morphology, migration, proliferation, cytoskeletal organization, and matrix marker expression, within a single integrated model.

We used this complex and validated model for high content phenotypic screening of small molecule compound libraries in 96 well-plate formats. For key compounds which performed well through the screen, we further evaluated the chondrogenic potential of the bioactive compounds, e.g., Trametinib, Panobinostat, SAHA, and Brefeldin A. We validated our compounds using a dose range with the expression of key chondrogenic markers SOX9, Collagen II, and aggrecan (Hardingh et al., 2006; Bonello et al., 2024) to confirm the chondrogenic effects of the compounds at the molecular level. This allowed us to better evaluate our selected compounds’ potential to induce chondrogenesis and enhance cell migration, both of which are critical for effective cartilage repair. We also explored actin texture analysis using SER-Spot and SER-Ridge indices to quantify changes in cytoskeletal organization, a critical factor in cellular differentiation and function (Treiser et al., 2010; Yourek et al., 2007).

This study provides important insights into the potential repurposing of cancer drugs such as MEK inhibitors with relevance to cartilage regeneration and a potential role in the treatment of musculoskeletal disease. The findings from this comparative study may open new therapeutic avenues for the treatment of cartilage-related diseases and offer a novel approach to enhancing tissue engineering strategies.

## 2 Materials and methods

### 2.1 3D GelMA cartilage model preparation

The 3D GelMA cartilage model was prepared based on a previously published protocol (Imere et al., 2024). Briefly, recombinant mouse Wnt3a protein (R&D Systems) was reconstituted to a concentration of 10 μg/mL stored at −80 °C in aliquots for up to 3 months. For experiments, working solutions of Wnt3a were prepared by diluting the stock to 600 ng/mL in Dulbecco’s Phosphate Buffered Saline (DPBS, Gibco). To immobilize Wnt3a on the surface of a 96-well plate, 40 µL of the working solution was added to each well, which had been pre-treated with a 2% (3-Aminopropyl)triethoxysilane (APTES, Merck) solution in 90% ethanol. Plates were incubated at room temperature (RT) for 30 min, followed by two washes with 100% ethanol. After incubation with Wnt3a for 1 h at RT, the wells were washed with DPBS.

Bone marrow stromal cells (BMSCs, Y201 line), generously provided by Prof. Paul Genever (University of York) (James et al., 2015), were maintained in growth medium [high-glucose DMEM was supplemented with 10% FBS, 1% L-glutamine, 1% penicillin-streptomycin (PS)] and routinely passaged every 2–3 days upon reaching ∼80% confluency. For chondrogenic differentiation, high-glucose DMEM was supplemented with 1% FBS, 1% L-glutamine, 1% penicillin-streptomycin (PS), 10% sodium pyruvate, 40 μg/ml L-proline, 50 μg/ml L-ascorbic acid-2-phosphate, 10% insulin-transferrin-selenium (ITS), 100 nM dexamethasone (all from Merck), and 10 ng/mL recombinant human TGF-β3 (Peprotech) as a positive chondrogenic medium. Basal medium consisted of high-glucose DMEM supplemented with 1% FBS, 1% L-glutamine, and 1% PS.

For the GelMA hydrogel synthesis, a 10% (w/v) solution was prepared by dissolving gelatin type A from porcine skin (300 bloom strength, Merck) in DPBS at 60 °C for 30 min based on published protocol (Barroso et al., 2022). Briefly, Methacrylic anhydride (MA, Merck) was added to the solution at a final concentration of 6% (v/v) and reacted for 2 h at 50 °C under vigorous stirring. Following the reaction, unreacted MA was partially removed by centrifugation at 3500 rpm for 5 min at RT. The solution was dialyzed against distilled water for 7 days at 37 °C using 12–14 kDa cut-off dialysis tubes (Thermo Scientific). After dialysis, the GelMA solution was diluted to 2% (w/v), and the pH was adjusted to 7.4 with 1 mM sodium hydroxide (Merck). The solution was then lyophilized for 2 days to produce a white porous foam, which was stored at −80 °C until further use.

To prepare the 4% (w/v) GelMA hydrogel precursor solution, the synthesized GelMA was dissolved in DPBS at 60 °C for 30 min and UV-sterilized for 5 min. Photoinitiator solutions of 40 mM riboflavin (Merck) and 546 mM sodium persulfate (Merck) were prepared in dH2O, filter-sterilized, and added to the GelMA solution at final concentrations of 2 mM and 10 mM, respectively. The prepared hydrogel precursor solution was stored in the dark until further use.

### 2.2 Set-up of the 3D GelMA cartilage model

Y201 cells were detached from culture dishes and resuspended in serum-free medium. The cells were seeded onto Wnt3a-immobilized 96-well plates at a density of 3 × 10^4^ cells per well using a MULTIDROP COMBI dispenser (Thermo Scientific) under a laminar flow hood. Cells were allowed to adhere for at least 1 h at 37 °C and 5% CO_2_. After the adhesion period, the medium was gently removed, and 50 µL per well of the prepared 4% (w/v) GelMA solution (containing the photoinitiators) was added using the same robotic dispenser. The GelMA solution was crosslinked by exposure to a 96-LED array (LUMIDUX II) for 10 min with varying light intensities (25, 35, 50, and 60 mW per well) to optimize the crosslinking conditions. Mechanical properties were characterized using Optical Coherence Elastography (OCE), following our previously published methodology (Imere et al., 2024). Mechanical contrast maps and Young’s modulus values were obtained to assess hydrogel stiffness across different light intensities (Supplementary Figures S1a,b). The 50 mW condition exhibited a significantly higher Young’s modulus compared to 35 mW and was similar to the optimized crosslinking conditions from our previous study (Imere et al., 2024). The selected GelMA stiffness range was chosen to support chondrogenic differentiation while permitting cell migration. The mechanical properties of the GelMA hydrogel are an important determinant of cell behavior and fate. Previous studies have demonstrated that matrix stiffness plays a critical role in regulating mesenchymal stem cell (MSC) expansion, migration, and lineage commitment, including chondrogenic differentiation. In particular, intermediate stiffness ranges have been shown to support rounded cell morphology, cytoskeletal reorganization, and enhanced production of cartilage-specific extracellular matrix components, whereas excessively soft or stiff matrices may impair differentiation, migration or promote alternative lineages (Imere et al., 2024; Seo et al., 2018). Based on these findings, the 50 mW intensity was selected for all subsequent experiments (Supplementary Figure S1b).

Once the hydrogels were set, 200 µL of the appropriate medium was added on top of each gel. The samples were incubated at 37 °C with 5% CO_2_ for 1 h to allow for further stabilization, after which the medium was replaced. For the basal control group (negative control, and compound screening, 190 µL of basal media was used, while chondrogenic media was used for the positive control samples. Media changes and compound treatments were performed using the VIAFLO 96 pipetting system (Integra).

### 2.3 Compound screening

In the initial phase of the study, we performed experiments to assess reproducibility across key experimental components, ensuring that variability remained below a predefined threshold [Coefficient of Variation (CV) < 20%), as defined by (Standard Dev ÷ Average x 100)] before proceeding with compound screening. The 3D GelMA cartilage model was set up as described earlier, with only basal media and chondro media samples. These samples were analyzed after 3 days of culturing, as shown in Supplementary Figure S2. For the compound screening phase, we utilized a bioactive drug library containing 55 compounds (listed in Supplementary Figure S3). A custom-designed bioactive small-molecule library comprising 55 compounds was assembled to interrogate key signaling pathways and cellular processes relevant to chondrogenesis, cytoskeletal organization, proliferation, stress responses, and matrix regulation. The library included kinase inhibitors (e.g., MEK, CDK, ROCK, Aurora, PI3K/mTOR), epigenetic modulators (HDAC inhibitors), cytoskeletal disruptors, proteasome and lysosomal inhibitors, apoptosis and caspase regulators, metabolic modulators, and clinically approved or investigational anticancer agents. This diverse functional coverage enabled unbiased phenotypic profiling across multiple regulatory axes implicated in cartilage development and degeneration.

The stock concentration for each compound is shown in the Supplementary Figure S3, but the final concentration used in the experiments was a 1000x dilution when added to the media. In the first step, all compounds, along with DMSO (final conc. Of 0.1% (v/v) used for the negative control samples), were diluted 50x in basal media (DMEM supplemented with 1% FBS, 1% L-glutamine, and 1% PS). From this diluted solution, 10 µL was added to 190 µL of cell culture medium (20x dilution), resulting in a final 1000x dilution of the compounds in the culture. Each compound was tested at three concentrations: undiluted (final concentration of 1000x dilution), 10x dilution (final concentration of 10000x dilution), and 100x dilution (final concentration of 100000x dilution), with two replicates per condition. Negative control wells (basal media) and positive control wells (chondrogenic media) were included in all experiments, as illustrated in Supplementary Figure S4. Compounds were diluted and added using the VIAFLO 96 pipetting system (Integra), and cells were stained, imaged, and analyzed after 3 days of treatment. Following the initial screening, selected compounds underwent further evaluation through a detailed dose-response study (Supplementary Figure S6). This phase involved testing eight different final concentrations as a semi-log dose response (0, 1, 3, 10, 30, 100, 300, and 1000 nM) for each selected compound, using the same experimental setup, with four replicates per concentration. To control for potential batch-to-batch variation, new batches of compounds were used. The experimental design is summarized in Supplementary Figures S6a,b.

### 2.4 Immunofluorescence staining

Samples were fixed in 4% paraformaldehyde for 1 h at room temperature and then washed with PBS. Following fixation, the samples were permeabilized and blocked simultaneously in a solution containing 2% bovine serum albumin (BSA) and 0.2% Triton-X in PBS for 2 h. For immunofluorescence staining, all antibodies and reagents were diluted in a 1% BSA/0.1% Triton-X solution and incubated for 2 h at room temperature. Cells were stained with Phalloidin-Atto 425 (Sigma) at a concentration of 1:100 to stain F-actin, HCS CellMask™ Deep Red Stain (ThermoFisher Scientific) at 1:5000 for cytoplasm staining (excitation range 650/655 nm), and DAPI (Merck) at 1:5000 for nuclear staining. After staining, the cells were washed three times with PBS-Tween wash buffer (500 mL PBS +500 µL Tween 20). All washing steps were conducted through a 405 Select Multiplate Washer (Biotek), and all solutions were dispensed using the MULTIDROP COMBI dispenser (Thermo Scientific). Finally, the samples were imaged using the Opera Phenix Plus High-Content Screening System (Revvity) to analyze cellular morphology and actin texture.

### 2.5 Cell imaging and morphology analysis

Cell morphology analysis was performed on images acquired using the Opera Phenix Plus High-Content Screening System (Revvity). Imaging was conducted in two distinct setups. In the first setup, plates were imaged in two channels, DAPI for nuclear staining and CellMask™ Deep Red for cytoplasm staining or three channels (DAPI, CellMask™ Deep Red, and Phalloidin-Atto) for dose response experiments with 10 fields selected per well and 8planes with step of 2 um using ×40 water objective (NA 1.1) captured from the bottom to provide a comprehensive 3D view of cellular morphology. In the second setup, used specifically for cell migration analysis, only the DAPI channel was used, and seven fields per well were imaged across 102 planes with step of 5 um usinig ×20 water objective (NA 1.0) to track nuclear movement and assess migration patterns. Image analysis was performed using both Harmony software (Revvity and Signal Image Artist (Revvity). These tools enabled automated detection and quantification of key cellular features, such as cellular size, shape, and cytoplasmic extension. A threshold was defined for cell area, with cells larger than 500 μm^2^ at any concentration flagged and selected for further analysis. Additionally, samples showing more than 200 cell migrations were selected for further evaluation, including dose-response analysis. These criteria allowed us to systematically identify and quantify significant morphological changes and migration behavior for downstream analysis.

### 2.6 Cell proliferation analysis

For cell proliferation analysis, the total number of nuclei was quantified in 3D images generated from 102 z-planes per well, using Signal Image Artist software (Revvity). The software allowed for accurate detection and counting of nuclei in the full z-stack, providing a comprehensive measure of cell density throughout the entire hight of GelMA. The total number of nuclei identified in each sample was taken as a direct representation of the total number of cells. This approach ensured precise evaluation of cell proliferation, as the number of nuclei corresponds to the number of cells in the 3D culture model.

### 2.7 3D cell migration analysis

For the 3D cell migration analysis, we first quantified the total number of nuclei that had migrated into specific z-planes within the 3D structure. Migration was assessed at the z-plane 20 (indicating 100 μm in depth), as well as at planes 40 (200 μm), 60 (300 μm), 80 (400 μm), and 100 (500 μm). The total number of migrated cells was calculated by summing the quantified nuclei from these five selected z-planes. This accumulated total was used as a representation of overall cell migration within the 3D model. All plane counts were performed on 3D images generated from a total of 102 z-planes per well, using the Signal Image Artist software (Revvity). In addition, Harmony software (Revvity) was used to create 3D reconstructed images of cell migration through the hydrogels, offering a visual representation of cellular movement within the gel matrix.

### 2.8 Actin texture analysis

For the Actin Texture Analysis, we used the Signal Image Artist software (Revvity), incorporating a module to calculate texture properties specifically on the Phalloidin-Atto 425 channel, which stains actin. The analysis was conducted using the SER (Structure Element Recognition) feature extraction method, set at a scale of 3 pixels with Kernel normalization. The actin texture was represented by two indices: SER-Spot, which highlights localized actin structures, and SER-Ridge, which emphasizes elongated or fibrous actin formations. These indices were used to evaluate the overall actin organization and structure within the cells.

### 2.9 Sox9 and collagen II expression study

To assess the chondrogenic effects of drugs in a simplified 2D assay, a 96-well plate experiment was conducted using the selected compound, Trametinib identified from the original GelMA model. Y201 cells were seeded at a density of 10,000 cells per well. Negative control wells were treated with Dulbecco’s Modified Eagle Medium (DMEM) supplemented with 1% Fetal Bovine Serum (FBS) and 1% Penicillin/Streptomycin/L-glutamine. Positive control wells were supplemented with factors known to stimulate chondrogenesis. Trametinib, diluted in DMSO, was added 24 h after seeding at varying concentrations, followed by incubation for an additional 9 days. After this period, cells were fixed with 10% neutral buffered formalin. Following fixation, samples were permeabilized and blocked with a solution of 2% bovine serum albumin (BSA) and 0.1% Triton-X in PBS for 2 h. Primary antibody incubation was conducted overnight at 4 °C using a goat anti-human SOX9 antibody (10 μg/mL, R&D Systems) and a rabbit anti-human Collagen II antibody (1:500 dilution, Sigma Aldrich). The next day, secondary antibodies were applied, including Alexa Fluor 568 donkey anti-goat (1:500 dilution, Invitrogen) and Alexa Fluor 488 donkey anti-rabbit (1:500 dilution, Invitrogen), alongside DAPI (1:5000 dilution, Merck) for nuclear staining. Plates were imaged at ×400 magnification using a Zeiss LSM 880 confocal microscope. Image analysis was performed with Fiji (ImageJ) to determine the percentage of SOX9-positive cells and quantify Collagen II expression.

### 2.10 qRT-PCR analysis of aggrecan expression in optimized trametinib treatment

To evaluate the expression of aggrecan (ACAN), a key component of the developing cartilage matrix, cells were treated with Trametinib (30 nM), a dose selected and optimized based on previous experiments to achieve maximal chondrogenic effects. A 2D culture assay was conducted using basal media (DMEM supplemented with 1% FBS and 1% Penicillin/Streptomycin/L-glutamine) as a control. After 7 and 14 days, total RNA was extracted using the RNeasy kit (Qiagen), and 500 ng of RNA was reverse-transcribed into cDNA using the High Capacity cDNA Reverse Transcription Kit (Applied Biosystems). Quantitative real-time PCR (qRT-PCR) was performed on the AriaMx Real-Time qPCR system using SYBR Green Master Mix (Thermo Fisher Scientific) and ACAN primers (Qiagen), following the manufacturer’s protocols. Gene expression was normalized to the housekeeping gene GAPDH, and fold changes were calculated using the comparative ΔCT method.

### 2.11 Statistical analysis

The number of replicates for each study is specifically mentioned in the respective sections. Data are expressed as mean ± standard deviation (SD). Statistical significance was assessed using one-way analysis of variance (ANOVA) followed by Tukey’s *post hoc* test for multiple comparisons across all experiments, including mechanical property measurements, cell morphology analyses (cell area and roundness), cell proliferation and migration analyses, actin texture analysis, and Collagen II signal quantification. In addition, an unpaired Student’s t-test was used for ACAN (aggrecan) gene expression analysis. A p-value of <0.05 was considered statistically significant. A significance panel is provided under all graphs, where comparisons between all groups are displayed, indicating the differences between each group.

## 3 Results

### 3.1 Establishing experimental consistency: minimizing variability in key parameters before bioactive compound screening

In the initial phase of the study, we conducted experiments to assess reproducibility of our chondrogenesis phenotypic assay across critical experimental components, including cell lines, hydrogel scaffolds, culture media, and antibody staining procedures. Our primary objective was to ensure that variability remained below a predefined threshold (CV < 20%) before proceeding to the compound screening phase. To achieve this, we adhered to optimized protocols for cell seeding, gel crosslinking, and media addition.

Reproducibility was evaluated by analyzing cellular morphology, with cell area and cell roundness revealed as key parameters. Coefficients of variation (%CVs) for these parameters were well below the maximal threshold (<20%). Specifically, the CV for cell area in basal media was 11%, compared to 18% in chondrogenic media. Similarly, the CV for cell roundness in basal media was 2.7%, while it was 3.1% in chondrogenic media (Supplementary Figure S2). These results confirmed that the experimental system had robust reproducibility, and it was suitable for advancing to the compound screening phase. The significant difference between basal and chondrogenic media further validated the model’s responsiveness to environmental conditions.

### 3.2 Screening of bioactive compounds reveals key modulators of chondrogenic morphology and cell migration

The subsequent phase of the study focused on screening a custom designed bioactive drug library. The library comprised 55 compounds. Each compound was tested in three different concentrations [i.e., undiluted (final concentration of 1000x dilution), 10x dilution (final concentration of 10000x dilution), and 100x dilution (final concentration of 100000x dilution)], with two replicates for each condition. The experimental setup also included negative control wells (basal media) and positive control wells (chondrogenic media). A detailed examination of cellular morphology was conducted across the drug plates to identify compounds that induced significant changes in cell shape and organization.

Analysis of heat maps (Supplementary Figure S4) highlighting the similarity between the effects of the compounds and those of chondrogenic media on key morphological parameters (such as cell area and cell roundness)enabled the identification of hit compounds across different concentration levels. Specifically, we identified Trametinib (100 nM, undiluted) and Panobinostat (100 nM, undiluted) as compounds significantly inducing chondrogenic-like morphology under undiluted conditions (P < 0.001). At a 10x dilution, Trametinib (10 nM) and SAHA (1000 nM) were observed to induce similar effects, while at a 100x dilution, Trametinib (1 nM) and Brefeldin A (10 nM) significantly induced chondrogenic-like morphology. Additionally, two compounds, Paclitaxel and Cytochalasin B, were observed to induce notable cell migration (P < 0.05).

Following these initial identifications, a closer examination of the selected compounds was conducted using statistical analysis to further assess their effects on cellular parameters. These parameters included cell morphology (cell area and roundness), cell proliferation (quantified by total nuclei count), and cell migration. The results demonstrated that the identified compounds not only mimicked chondrogenic media in inducing chondrocyte-like morphology but also modulated cell migration and proliferation in distinct ways depending on the compound and concentration tested.

### 3.3 Morphological analysis of cell area and roundness for selected compounds

**FIGURE 1:**
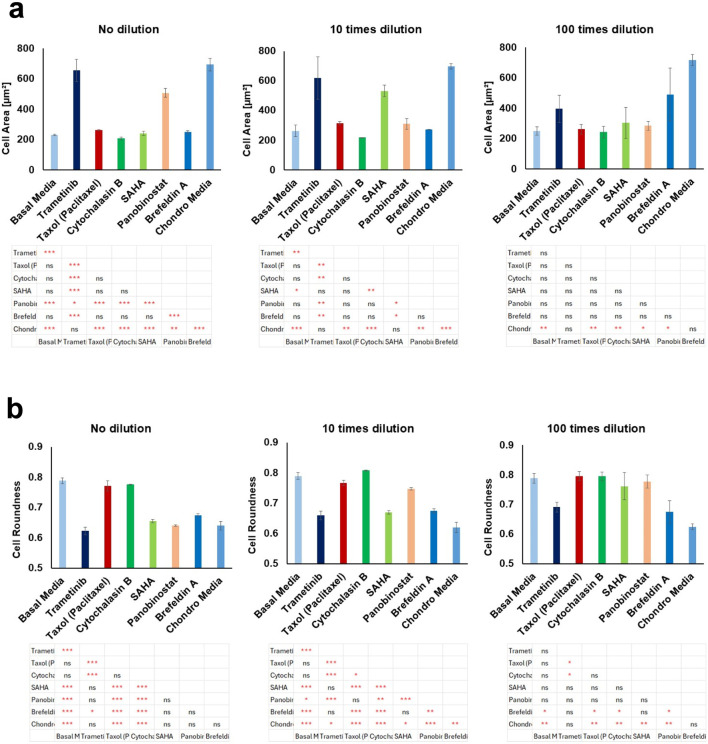
Effects of selected bioactive compounds on cell morphology. **(a)** Analysis of cell area for different compounds at three concentration levels (undiluted, 10x dilution, and 100x dilution). **(b)** Cell roundness for the same compounds and concentrations. Data are expressed as mean ± SD, with statistical significance indicated by *p < 0.05; **p < 0.01; ***p < 0.001; ns: not significant.

**FIGURE 2:**
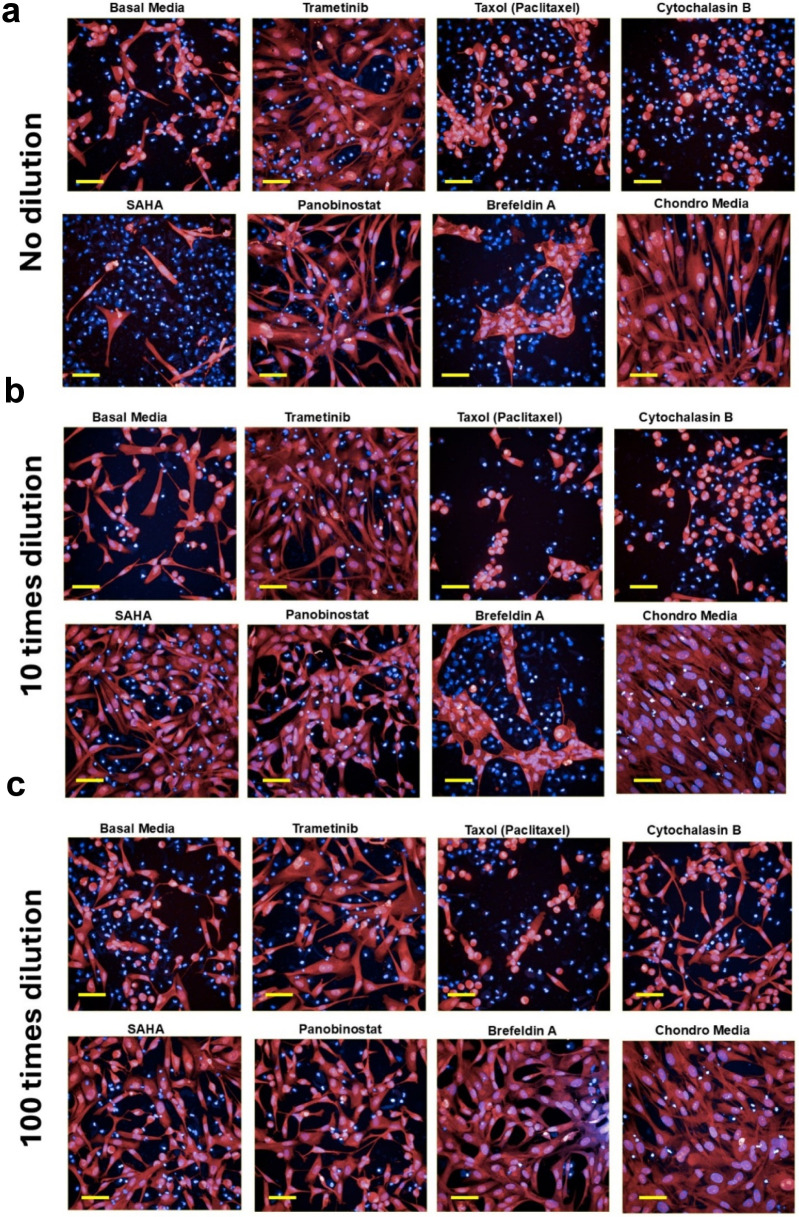
Fluorescent images of samples treated with bioactive compounds, stained with DAPI (nuclei -blue) and CellMask (cytoplasm -red) at different compound concentration levels: **(a)** Undiluted, **(b)** 10x dilution, and **(c)** 100x dilution. These images highlight the cellular distribution and morphology under various treatment conditions. Scale bars = 50 µm.

To investigate the effects of the selected bioactive compounds on cell morphology, we analyzed the cell area and roundness indices under different experimental conditions (Figures 1, 2). At the non-diluted concentration level, where Trametinib and Panobinostat were both tested at 100 nM, these compounds exhibited significant changes in cell morphology (P < 0.001). Specifically, the average cell area for Trametinib was 656.7 µm^2^, and for Panobinostat, it was 508.1 µm^2^, both of which were significantly larger than the cell area of basal media samples (231.6 µm^2^, P < 0.001). These values were also very similar to the cell area observed in chondrogenic media-treated samples (694.4 µm^2^), indicating that Trametinib and Panobinostat induced chondrogenic-like expansion of cells at this concentration. The remaining compounds tested at this concentration exhibited cell areas comparable to basal media, showing limited chondrogenic-like activity. The same trend was observed in the analysis of cell roundness. For the roundness index, Trametinib and Panobinostat demonstrated values of 0.62 and 0.64, respectively, which closely matched the roundness index of cells in the chondrogenic media condition (0.64). In contrast, the roundness index in the basal media-treated cells was significantly higher at 0.78, indicating that these cells maintained a more rounded morphology (P < 0.001).

At 10x dilution, Trametinib at 10 nM maintained its chondrogenic-like impact, with an average cell area of 618.1 µm^2^, closely resembling the cell area in chondrogenic media. SAHA at 1000 nM also showed a significant increase in cell area, with an average of 532 μm^2^, again notably larger than the cell area in basal media (P < 0.001) and comparable to chondrogenic media. In terms of cell roundness, Trametinib and SAHA maintained similar effects to those observed at the undiluted concentration. Trametinib had a roundness index of 0.65, while SAHA exhibited a roundness index of 0.66, both of which were comparable to chondrogenic media.

At 100x dilution, Trametinib at 1 nM continued to show a moderate effect on increasing cell area, although this change was not statistically significant. However, Brefeldin A at 10 nM exhibited a notable effect on cell morphology, significantly increasing cell area compared to basal media. Additionally, Brefeldin A induced a significant reduction in the cell roundness index, reaching 0.67, which was significantly lower than the roundness index in basal media (P < 0.05), suggesting that Brefeldin A may also promote aspects of chondrogenic-like differentiation, although to a lesser extent than Trametinib and SAHA at higher concentrations. Overall, the compounds Trametinib, Panobinostat, SAHA, and Brefeldin A demonstrated significant, concentration-dependent effects on cell area and roundness, indicating their potential role in promoting chondrogenesis or altering cell morphology in a manner consistent with chondrogenic differentiation.

### 3.4 Cell proliferation analysis for selected compounds

**FIGURE 3:**
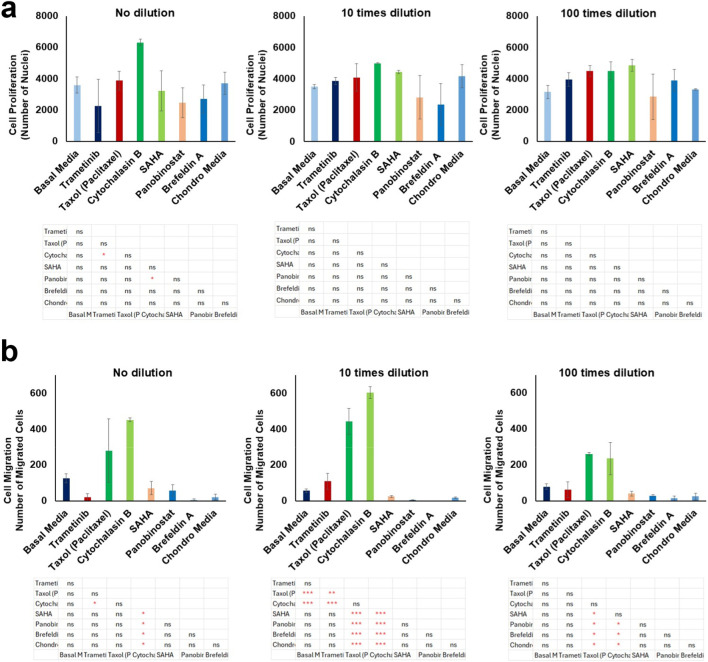
Effects of selected bioactive compounds on cell proliferation and cell migration. **(a)** Analysis of cell proliferation for different compounds at three concentration levels (undiluted, 10x dilution, and 100x dilution). **(b)** Cell migration for the same compounds and concentrations. Data are expressed as mean ± SD, with statistical significance indicated by *p < 0.05; **p < 0.01; ***p < 0.001; ns: not significant.

We assessed cell proliferation in the different experimental conditions by counting and calculating the total number of nuclei from 3D images (Supplementary Figure S5), which included both migrated and non-migrated cells. Our data did not show any significant changes in the number of nuclei between the selected compounds, the negative control, and the positive control, as reflected in the statistical analysis presented in the graph (Figure 3a). The only significant observation was a lower number of nuclei with Panobinostat and Trametinib compared to Cytochalasin B (P < 0.05). This suggests that Trametinib and Panobinostat did not significantly increase cell number.

### 3.5 Cell migration analysis for selected compounds

**FIGURE 4:**
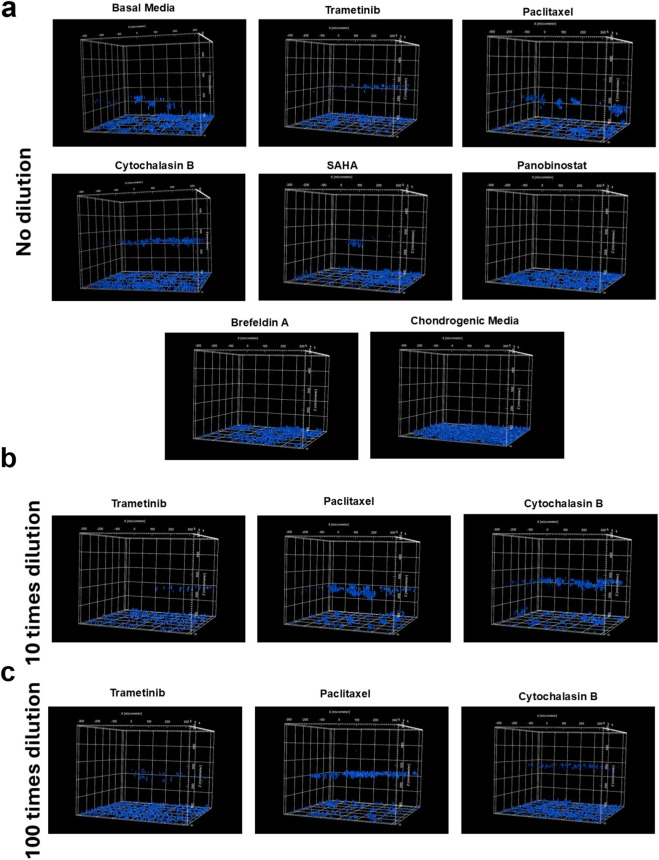
Representative fluorescence 3D images of cells after imaging the DAPI channel across 102 z-planes with 5 µm intervals, showing cell migration in response to bioactive compounds at different concentration levels: **(a)** undiluted, **(b)** 10x dilution, and **(c)** 100x dilution. These images illustrate the extent of cell migration within the 3D structure under varying treatment conditions.

We investigated cell migration by calculating the total number of cells that migrated across different Z-planes within the hydrogel (100, 200, 300, 400, and 500 µm). The data indicated that, in general, cells in basal media exhibited more migration compared to those in chondrogenic media (Figures 3b, 4). At the non-diluted concentration, Cytochalasin B (3 µM) significantly induced cell migration (P < 0.05). Additionally, Paclitaxel (0.3 µM) also showed an increase in the number of migrating cells; however, due to high variability between samples, this increase was not statistically significant.

At the 10x dilution level, Cytochalasin B (300 nM) and Paclitaxel (30 nM) induced a significant increase in cell migration (P < 0.001) compared to both basal and chondrogenic media, as well as to their non-diluted forms. Interestingly, among the compounds that promoted chondrogenic-like morphology, Trametinib (10 nM) at this dilution level also showed a notable increase in cell migration, suggesting a dual role in both promoting chondrogenesis and enhancing cell motility, which is particularly intriguing. At 100x dilution, Cytochalasin B (30 nM) and Paclitaxel (3 nM) continued to show significant effects on cell migration compared to other compounds (P < 0.05). However, their impact was reduced compared to their 10x concentrated samples, indicating a dose-dependent response where the 10x dilution level exhibited the strongest induction of cell migration.

### 3.6 Dose-response analysis of selected compounds

Following the initial screening and selection of compounds, we conducted a detailed dose-response study to further evaluate their effects. This phase involved testing eight different concentrations (0, 1, 3, 10, 30, 100, 300, and 1000 nM) for each selected compound, using the same experimental setup as the previous phase, with four replicates per concentration. To account for any potential batch-to-batch variation, all compounds were sourced from new batches. The experimental design is summarized in Supplementary Figures S6a,b.

In this dose-response analysis, we not only continued to assess the key morphological parameters, including cell area and cell roundness, but also introduced additional measurements focusing on the texture analysis of actin staining. This was performed using two key indices: SER-Spot and SER-Ridge, which quantify specific aspects of cytoskeletal organization and texture patterns in the cells. These additional metrics provided deeper insight into the compounds’ effects on cellular architecture and cytoskeletal remodeling.

### 3.7 Morphological analysis of dose-response in selected compounds

**FIGURE 5:**
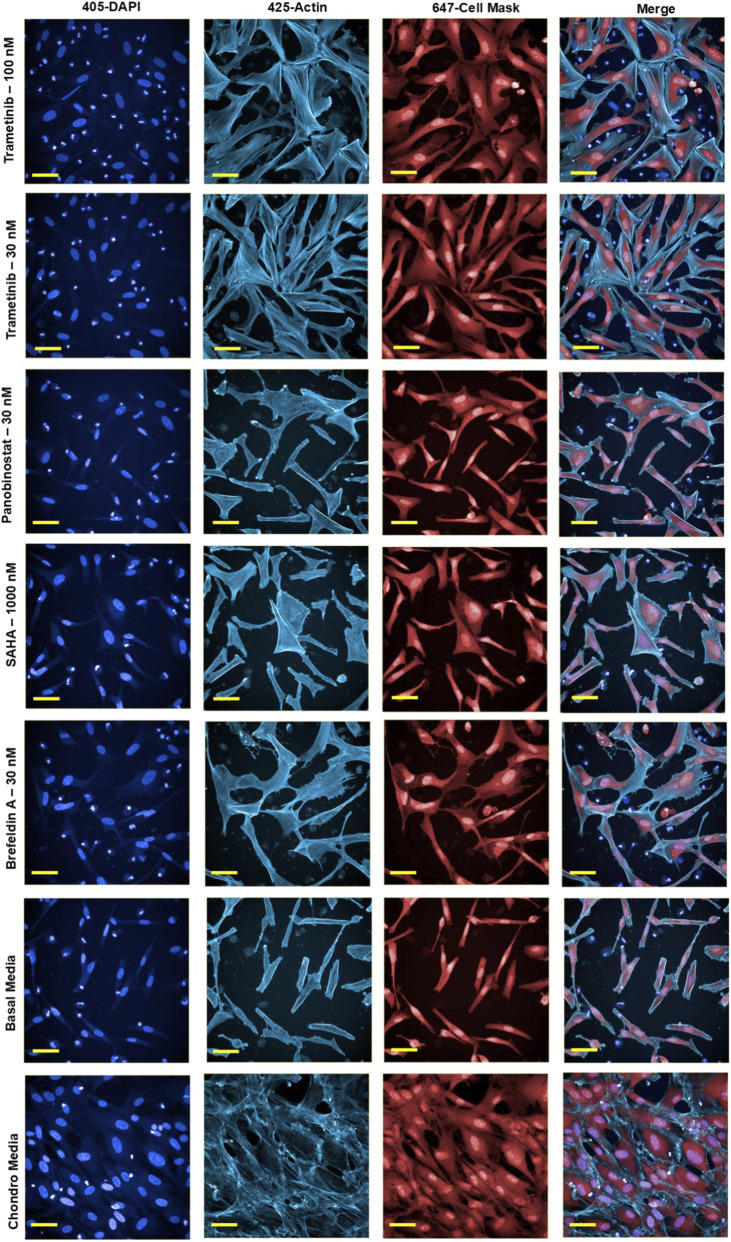
Fluorescent images of samples treated with bioactive compounds, stained with DAPI (nuclei) and CellMask (cytoplasm), Phalloidin-Atto 425 (Actin) at selected concentration levels. These images highlight the cellular distribution and morphology in addition to actin texture under various treatment conditions. Scale bars = 50 µm.

The dose-response analysis of selected compounds across eight different concentrations revealed significant effects on cell morphology, particularly cell area and roundness. Trametinib, at concentrations ranging from 3 nM to 1000 nM, significantly increased cell area compared to basal media samples (P < 0.01). Among these, the concentrations of 10 nM, 30 nM, and 100 nM exhibited the most pronounced increase in cell area, closely resembling the values observed in chondrogenic media samples. For other compounds, specific concentrations induced changes in cell morphology similar to chondrogenic conditions: Panobinostat at 30 nM, SAHA at 1000 nM, and Brefeldin A at 30 nM all significantly increased cell area compared to basal media, while simultaneously reducing cell roundness to levels comparable to chondro media (P < 0.001) (Figure 5; Supplementary Figures S6c,d, S7, S8). These findings align well with the previous screening results obtained with the broader compound library tested at three different concentrations, further supporting their chondrogenic potential. In contrast, both Cytochalasin B and Paclitaxel did not induce any morphological changes that mimicked chondro media at any concentration, consistent with our previous observations during the compound screening phase. This consistency across multiple phases of the experiment, even with newly purchased batches of compounds, validates the reproducibility of our findings and underscores the robustness of the data across different experimental setups.

### 3.8 Actin texture analysis of dose-response in selected compounds

**FIGURE 6:**
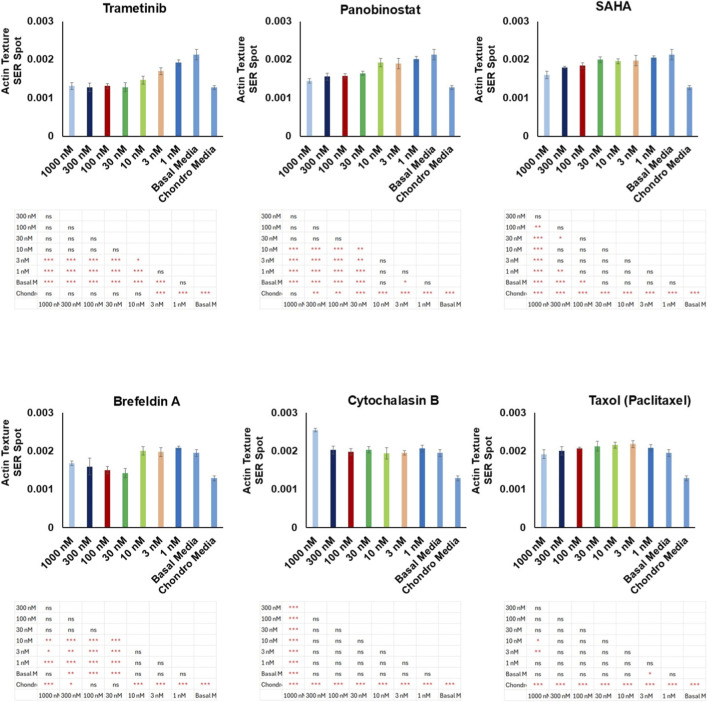
Effects of selected bioactive compounds on actin texture analysis through the SER spot indice at eight concentration levels (0, 1, 3, 10, 30, 100, 300, and 1000 nM). Data are expressed as mean ± SD, with statistical significance indicated by *p < 0.05; **p < 0.01; ***p < 0.001; ns: not significant.

We further examined the effects of the selected compounds on actin cytoskeletal organization using texture analysis quantified by two indices: SER-Spot and SER-Ridge (Supplementary Figures S6e,f). These indices assess specific aspects of actin patterning and cytoskeletal texture in the cells. The analysis revealed that Trametinib, at concentrations of 10 nM, 30 nM, and 100 nM—where the compound also showed promising results in cell morphology—significantly altered actin texture compared to basal media (P < 0.001). Importantly, there was no significant difference in the actin texture of these samples compared to chondro media, further confirming that Trametinib induces cellular changes closely resembling those seen under chondrogenic conditions (Figure 6; Supplementary Figure S9).

In addition, for Panobinostat at 30 nM, SAHA at 1000 nM, and Brefeldin A at 30 nM, significant changes in actin texture were observed (P < 0.001). These compounds exhibited some degree of similarity in SER-Spot and SER-Ridge values compared to chondro media, indicating partial alignment with chondrogenic actin organization (Figure 6; Supplementary Figure S9). This suggests that although these compounds induce some level of cytoskeletal reorganization, their effects on actin patterning are distinct from the fully chondrogenic-like response seen with Trametinib. These findings add another layer of evidence for the differential effects of these bioactive compounds on cell morphology and cytoskeletal architecture. A summary of the effects of the selected bioactive compounds on morphology, migration, proliferation, and cytoskeletal organization is provided in Supplementary Table S1.

### 3.9 SOX9, collagen II, and aggrecan expression analysis

**FIGURE 7:**
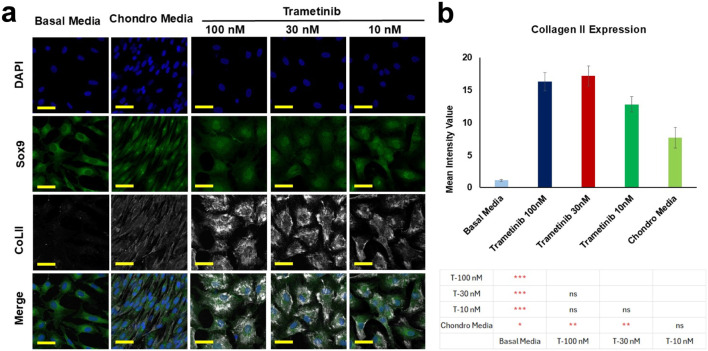
**(a)** Fluorescent images of samples treated with Trametinib, stained with DAPI (blue), SOX9 (green), and Collagen II (gray) at selected concentration levels (10, 30, and 100 nM). Scale bars = 50 µm. **(b)** Quantification of fluorescent Collagen II signals from image analysis. Data are expressed as mean ± SD, with statistical significance indicated by *p < 0.05; **p < 0.01; ***p < 0.001; ns: not significant.

Among the screened compounds, Trametinib was prioritized for further analysis due to its consistent performance across multiple phenotypic endpoints. In the final phase of the study, we investigated two key markers of chondrogenesis—SOX9 and Collagen II—in response to Trametinib at concentrations of 10 nM, 30 nM, and 100 nM, using a 2D culture model. The data presented in Figure 7 demonstrate that, while the expression levels of SOX9 between samples were not significantly different across conditions, the expression of Collagen II was significantly increased in the presence of Trametinib compared to chondro media samples in particular in the concentration of 100 nM and 30 nM (P < 0.01). Notably, this increase occurred without the addition of traditional chondrogenic factors, highlighting the unique chondrogenic potential of Trametinib. This finding provides further confirmation of Trametinib’s ability to drive chondrogenic differentiation and suggests its potential as a therapeutic agent for cartilage-related diseases.

Gene expression analysis of Aggrecan (ACAN) was also conducted to assess chondrogenesis following treatment with the optimized dose of Trametinib (30 nM) on day 7 and day 14 (Supplementary Figure S10). The results demonstrated a significant upregulation of aggrecan expression, with a 13.6-fold increase on day 7 and a 21.4-fold increase on day 14, compared to the control group (P < 0.05).

## 4 Discussion

The results of initial development of our chondrogenesis high content phenotypic screening assay confirmed that the experimental system had minimal variability and was suitable for advancing to the compound screening phase. Automated liquid handling robotic systems play a crucial role in maintaining consistency throughout these experiments with our complex models. Automated systems were employed for tasks such as cell seeding, gel placement in 96-well arrays, and gel crosslinking. Liquid handling robots also added compounds to the wells, fixed cells, and carried out the staining and washing processes. This automation significantly minimized human error and reduced potential sources of variability, thereby enhancing the reliability of the data.

The findings of the 55 compound screening study provide valuable insights into the chondrogenic potential of selected bioactive compounds, particularly Trametinib, Panobinostat, SAHA, and Brefeldin A, within the context of 3D cartilage models. By evaluating changes in cell morphology, proliferation, migration, cytoskeletal reorganization, and the expression of key chondrogenic markers such as SOX9 and Collagen II, this study offers a comprehensive understanding of how these compounds can promote or modulate chondrogenesis. The use of 3D hydrogel scaffolds, which better mimic the native extracellular environment of cartilage, enhances the relevance of these findings for potential therapeutic applications in cartilage repair and regeneration.

The observed changes in cell morphology, particularly the increase in cell area and the reduction in cell roundness induced by chondrogenic media compared to basal media are consistent with previous studies (Green et al., 2015; Ichinose et al., 2010; Ichinose et al., 2005; Ichinose et al., 2013). These studies have demonstrated that cell spreading and elongation are key features associated with early-stage chondrogenic differentiation. During the initial phase of chondrogenesis, typically between days 3–7, mesenchymal stem cells (MSCs) often adopt a more elongated, spindle-like shape when cultured in 3D environments such as pellets (Ichinose et al., 2010; Ichinose et al., 2005) or hydrogels (Ichinose et al., 2013). This transformation marks their transition to chondroprogenitor cells. In later stages (starting from 2 weeks of culture) MSCs undergo further differentiation, becoming more rounded as they mature into chondrocytes. Additionally, the increase in cell size is a hallmark of chondrogenic differentiation, with mature chondrocytes eventually becoming hypertrophic during terminal differentiation and maturation (Green et al., 2015). Given these well-established morphological changes, it is expected that cells cultured in chondrogenic media for 3 days would exhibit the characteristic spindle-like elongation and increased cell area, indicative of their progression toward a chondroprogenitor state. These changes in cell morphology are in line with the early events in the chondrogenic differentiation pathway (Green et al., 2015; Zha et al., 2021).

The observed changes in cell morphology, particularly the increase in cell area and the reduction in cell roundness induced by Trametinib and Panobinostat, SAHA and Brefeldin A suggest that these compounds promote a chondroprogenitor-like phenotype in mesenchymal stem cells (MSCs). The significant increase in cell area seen with Trametinib at 100 nM and 10 nM, can be through the MEK inhibition (Attwood et al., 2021; U.S.F. and D. Administration, 2018), a primary action of Trametinib, can promote chondrocyte differentiation by suppressing the ERK pathway (Wang et al., 2018; Stanton et al., 2003). This finding is further supported by the observation that the cell roundness induced by Trametinib closely resembles that observed in chondrogenic media. In the screening study, it has been shown that Panobinostat at a concentration of 100 nM, and SAHA (vorinostat) at the concentration of 1000 nM significantly altered the morphology of MSCs, closely resembling the changes seen in chondro media-treated samples. This suggests that these compounds can induce chondrogenic-like transformations in MSCs, potentially through their action as histone deacetylase inhibitors (HDACi) (Parveen et al., 2023; Di Bello et al., 2022; Wan et al., 2021), which regulates gene expression involved in chondrogenesis (Hong et al., 2009; Lawlor et al., 2019; Meng et al., 2018). By inhibiting HDAC activity, Panobinostat and SAHA increase the acetylation of histones, leading to the activation of key chondrogenic genes which are essential for early chondroprogenitor cell differentiation (Wan et al., 2021; Hong et al., 2009). Furthermore, SAHA (vorinostat) inhibits the production of various matrix metalloproteinases (MMPs) and nitric oxide (NO) induced by IL-1β in human chondrocytes by blocking p38 and MEK-ERK signaling pathways (Zhong et al., 2013). Since inhibition of MEK-ERK signaling is known to enhance chondrogenesis (Wang et al., 2018; Stanton et al., 2003), SAHA’s action on this pathway likely contributes to its ability to promote chondrogenic differentiation, further supporting the chondroprogenitor-like phenotype and contributing to the observed morphological changes. Brefeldin A also induced significant morphological changes at lower concentrations (10 nM), particularly in terms of reducing cell roundness. This suggests that Brefeldin A may also play a role in promoting chondrogenic differentiation, possibly through its effects on Golgi apparatus function and protein trafficking (Ilya et al., 2021; Dejgaard and Presley, 2020). However, the extent of these changes was not as pronounced as those induced by Trametinib, Panobinostat, and SAHA indicating a potentially weaker or more indirect chondrogenic effect.

The analysis of actin texture, using SER-Spot and SER-Ridge indices, further confirmed the chondrogenic potential of the selected screened compounds by demonstrating similar cytoskeletal organization to that observed in chondro media samples. Cytoskeletal organization, particularly actin filament dynamics, is known to play a crucial role in cellular differentiation processes, including chondrogenesis (Venit et al., 2021; Saidova and Vorobjev, 2020). Early studies have demonstrated that osteoblasts exhibit higher stiffness compared to MSCs and chondrocytes (Yourek et al., 2007; Saidova and Vorobjev, 2020; Darling et al., 2008), supporting the hypothesis that a reduction in cellular rigidity (following the depolymerization of actin filaments) promotes adipogenesis or chondrogenesis, while increased actin polymerization encourages the osteogenic differentiation of MSCs (Pablo Rodríguez et al., 2004; Sonowal et al., 2013; Lim et al., 2000). This suggests that cellular mechanical properties, particularly the dynamics of the actin cytoskeleton, play a critical role in determining lineage commitment. Therefore, manipulating actin filament organization could provide a means of guiding MSCs toward chondrogenic differentiation, enhancing their potential for cartilage regeneration (Saidova and Vorobjev, 2020; Lim et al., 2000). The significant alterations in actin texture induced by Trametinib at 10 nM and 30 nM suggest that this compound promotes cytoskeletal reorganization in a manner conducive to chondrogenic differentiation. This finding is consistent with previous studies that have linked changes in actin dynamics with stem cell fate decisions, including the promotion of chondrogenesis.

Panobinostat and SAHA also showed some degree of cytoskeletal reorganization, as evidenced by changes in SER-Spot and SER-Ridge values. However, their effects were less pronounced compared to Trametinib, suggesting that while these compounds can induce partial chondrogenic-like changes in cytoskeletal architecture, they may not be as potent as Trametinib in driving full chondrogenic differentiation. These differences in cytoskeletal reorganization likely reflect the distinct molecular targets of these compounds. While Trametinib directly inhibits the MEK/ERK pathway, Panobinostat and SAHA are histone deacetylase (HDAC) inhibitors, which modulate gene expression through epigenetic mechanisms rather than directly affecting cytoskeletal components.

Paclitaxel and Cytochalasin B significantly promoted cell migration; however, their limited impact on inducing chondrogenic morphology and marker expression suggests that their primary effect lies in enhancing cell motility rather than driving chondrogenic differentiation. Paclitaxel stabilizes microtubules (Prota et al., 2023; Nasrin et al., 2020), facilitating the formation of cellular protrusions needed for migration, but it does not appear to activate key pathways involved in chondrogenesis. Similarly, Cytochalasin B disrupts actin filaments, (Kim H. G. et al., 2023), reducing cellular rigidity and increasing migration by altering cytoskeletal dynamics, but this disruption may inhibit the organized cytoskeletal changes required for mesenchymal stem cells (MSCs) to commit to a chondrogenic lineage. Thus, while both compounds effectively boost motility, they lack the signaling modulation necessary for chondrogenic differentiation. In contrast, Trametinib showed a dual effect of promoting chondrogenic-like morphology while also enhancing cell migration, particularly at 10 nM. The ability to enhance both differentiation and migration is critical for effective cartilage repair, as cell migration is essential for repopulating damaged tissue and promoting tissue regeneration. This dual effect is particularly intriguing, as most compounds tend to focus on either differentiation or migration, but not both.

The significant increase in Collagen II expression induced by Trametinib, particularly at 100 nM and 30 nM, provides molecular confirmation of its chondrogenic potential. Collagen II is a critical extracellular matrix component of cartilage, and its upregulation is a hallmark of chondrogenic differentiation (Shah and Mithoefer, 2021; Peng et al., 2021). Furthermore, the observed upregulation of aggrecan expression following treatment with Trametinib (30 nM) highlights the compound’s potential to promote chondrogenesis. The significant fold increases on day 7 (13.6-fold) and day 14 (21.4-fold) indicate a sustained and progressive enhancement of cartilage-specific matrix production over time. The increase in collagen II and aggrecan expression observed with Trametinib might result from MEK/ERK inhibition (Attwood et al., 2021; U.S.F. and D. Administration, 2018), a primary action of Trametinib, which is known to promote chondrocyte differentiation and matrix production (Wang et al., 2018; Stanton et al., 2003). Interestingly, SOX9 expression did not show significant changes across conditions, which may suggest that Trametinib primarily enhances the later stages of chondrogenesis, where matrix production (e.g., Collagen II and aggrecan) becomes more prominent, rather than the early commitment phase typically marked by SOX9 upregulation (Zhang et al., 2021; Song and Park, 2020). Current regenerative approaches often rely on growth factors, biomaterial scaffolds, or cell-based therapies, each of which provides complementary biochemical and mechanical signals. Inhibition of the MEK/ERK pathway has been shown to promote chondrogenic differentiation and matrix production, suggesting that MEK inhibitors could synergize with pro-chondrogenic growth factors or mechanically optimized scaffolds to enhance cartilage regeneration. While combinatorial effects were not directly assessed in the present study, our findings provide a strong rationale for future investigations exploring MEK pathway modulation alongside established regenerative cues, particularly in advanced 3D or osteochondral models.

## 5 Conclusion

This study demonstrates the potential of repurposing small-molecule inhibitors which modulate the MEK pathway, particularly Trametinib, for cartilage regeneration and repair. By promoting both chondrogenic differentiation and cell migration, Trametinib emerges as a promising candidate for future therapeutic applications. The findings also highlight the importance of combining multiple assays and phenotypic readouts, including morphological, cytoskeletal, and molecular analyses, to comprehensively evaluate the chondrogenic potential of bioactive compounds. Future studies should focus on *in vivo* validation and the development of target delivery systems to maximize the therapeutic efficacy of Trametinib and other compounds identified in this study. In addition, our novel chondrogenesis high content screening assay could be employed for higher throughput screening with larger libraries of FDA approved drugs, annotated chemical-genetic sets and diverse chemical libraries could be screened to identify additional drug repurposing opportunities, therapeutic targets and chemical starting points respectively for novel drug discovery programs targeting cartilage regeneration and repair.

## Data Availability

The raw data supporting the conclusions of this article will be made available by the authors, without undue reservation.
