# Supplementary material for: High-throughput 3D phenotypic screening identifies repurposed MEK inhibitors as drivers of chondrogenesis for cartilage regeneration
Source: Front Bioeng Biotechnol. 2026 Feb 23;14:1748443. doi: 10.3389/fbioe.2026.1748443 (PMC12968188; doi:10.3389/fbioe.2026.1748443)
Supplement: Supplementary file 1 [file Supplementaryfile1.docx]

**Supporting Information**

**High-throughput 3D phenotypic screening identifies repurposed MEK inhibitors as drivers of chondrogenesis for cartilage regeneration**

Hadi Hajiali^1*^, Justyna Cholewa-Waclaw^2^, Jacob Ballard^1^, Kerime Ebrar Okur^1^, Richard Elliott^3^, Neil O Carragher^3^, Alicia J. El Haj^1*^

*^1^Healthcare Technologies Institute, Institute of Translational Medicine, School of Chemical Engineering, University of Birmingham, Birmingham, United Kingdom.*

*^2^Institute for Regeneration and Repair, University of Edinburgh, Edinburgh, United Kingdom.*

*^3^Cancer Research UK Scotland centre, Institute of Genetics and Cancer, University of Edinburgh, Edinburgh, United Kingdom.*

*Corresponding authors: [h.hajiali@bham.ac.uk](mailto:h.hajiali@bham.ac.uk); [a.elhaj@bham.ac.uk](mailto:a.elhaj@bham.ac.uk)


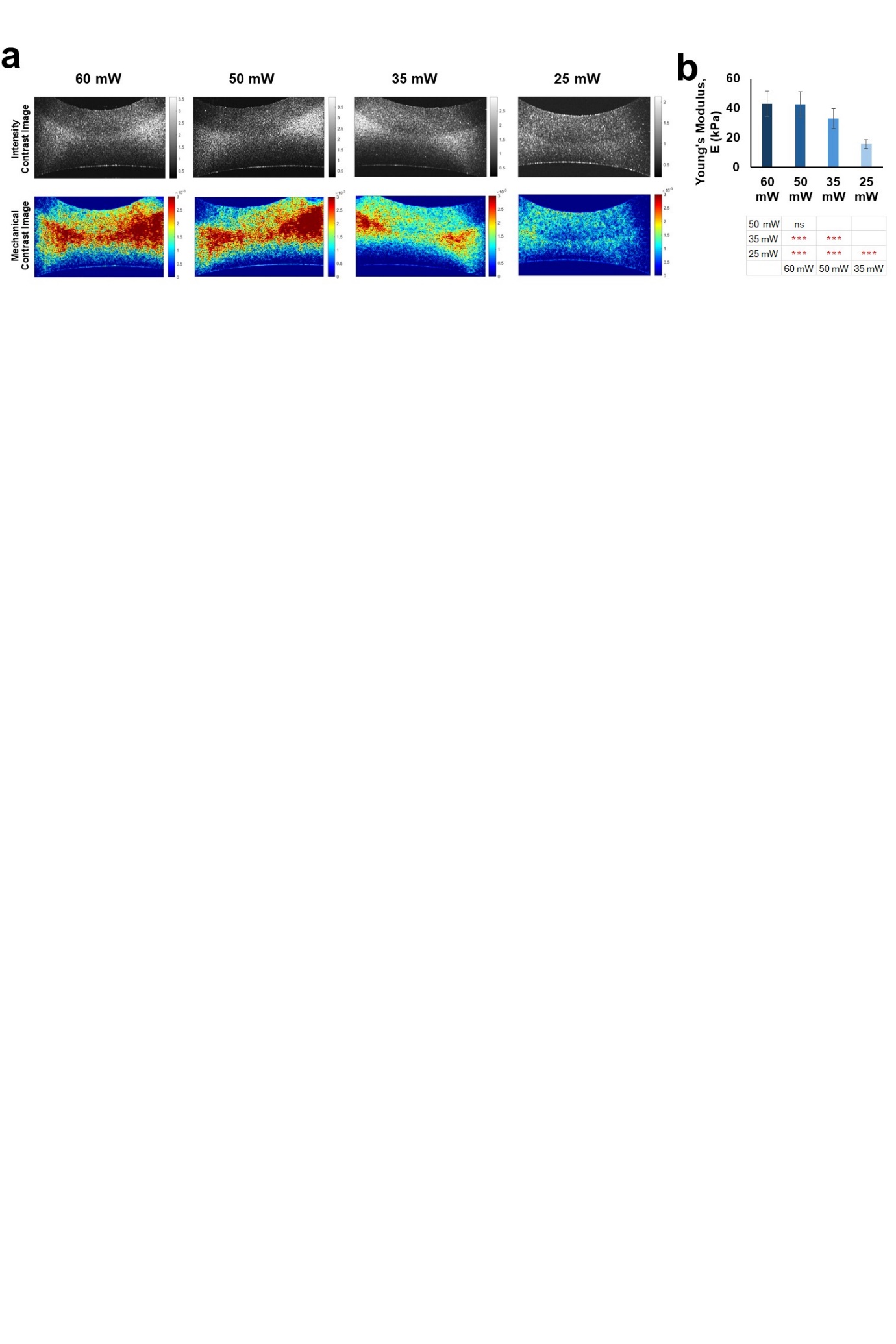


**Figure S1.** Mechanical property analysis of GelMA hydrogels crosslinked using different LED light intensities. (a) Depth-resolved images showing intensity contrast (top) and spatial mechanical contrast (bottom), presented as heatmaps. (b) Calculated Young’s modulus values for each experimental group. Data are expressed as mean ± SD, with statistical significance indicated by *p<0.05; **p<0.01; ***p<0.001; ns: not significant.


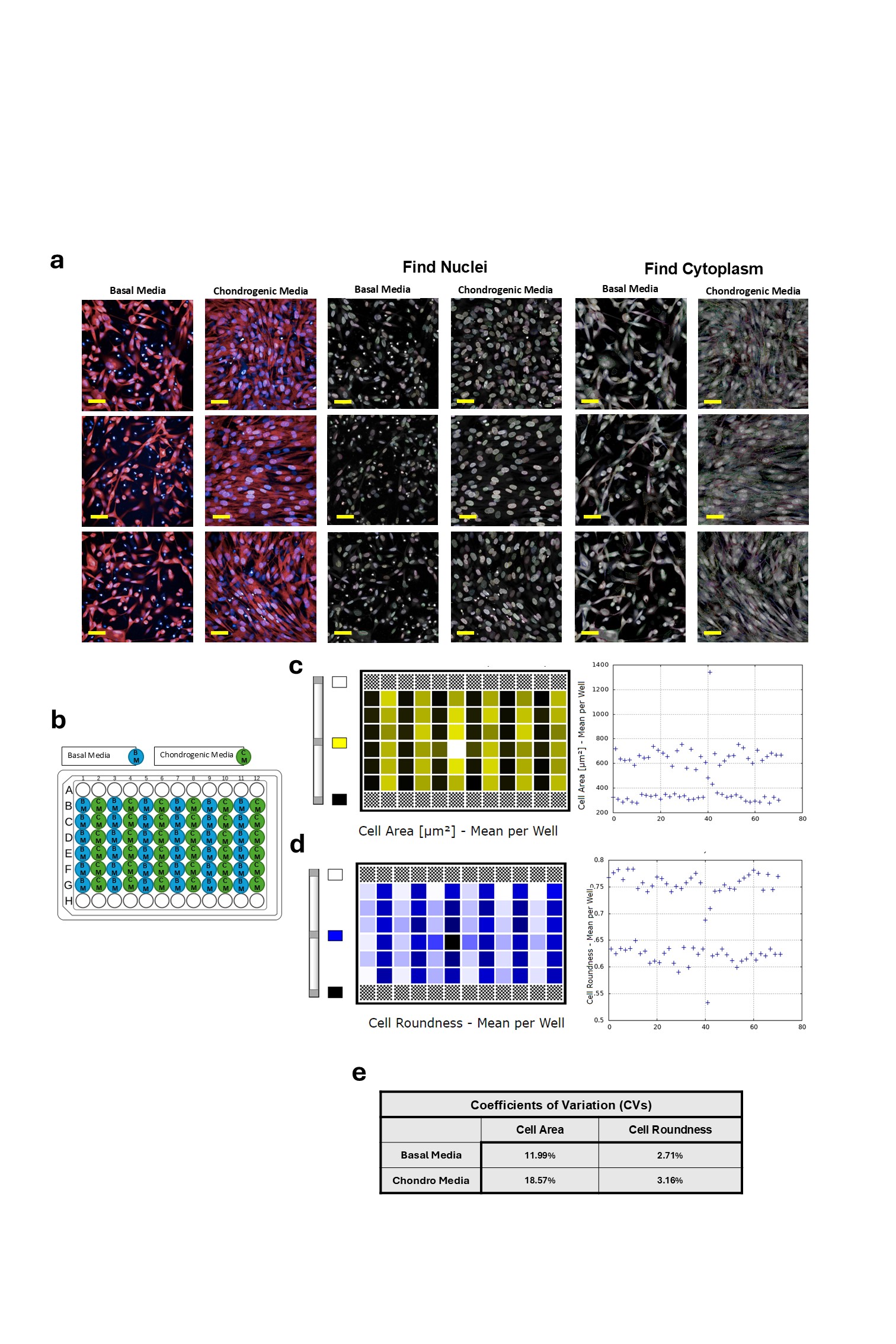


**Figure S2**. (a) Fluorescent images of samples treated with basal media and chondrogenic media, stained with DAPI (nuclei) and CellMask (cytoplasm). These images highlight cellular distribution and morphology under both conditions, and show the segmentation of images to identify nuclei and cytoplasm using Signal Image Artist software. Scale bars = 50 µm. (b) Schematic design of the 96-well plate layout, including sample placements. (c) Effects of basal and chondrogenic media on cell area. (d) Effects of basal and chondrogenic media on cell roundness. (e) Coefficient of Variation (CV) calculated for the cell area and cell roundness in different group of samples.


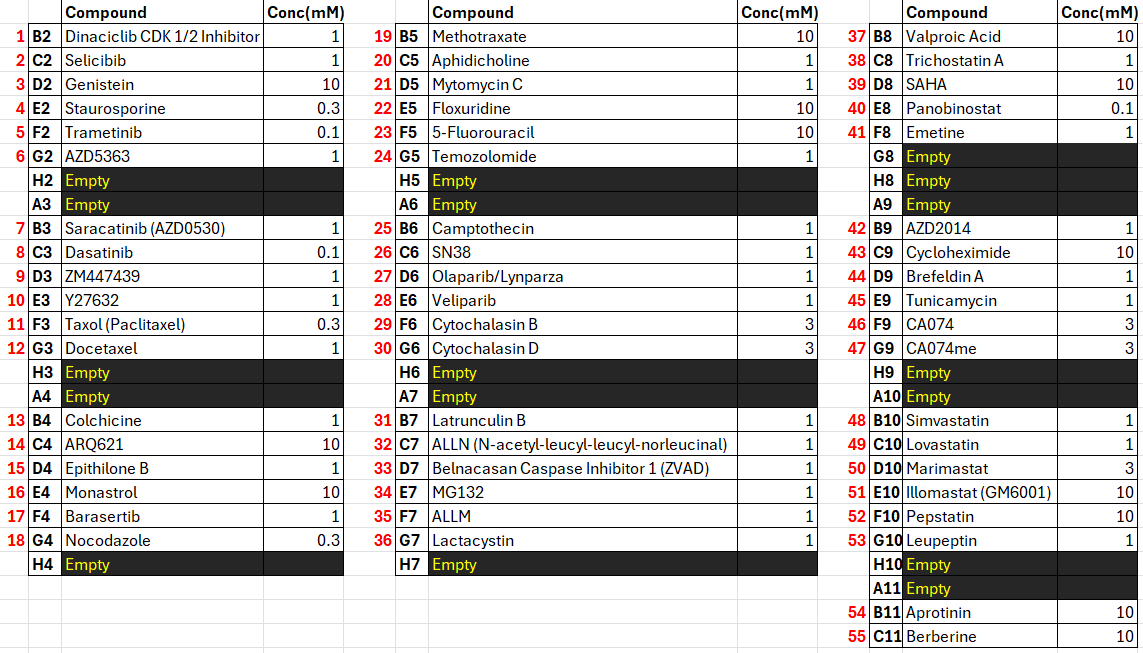


**Figure S3**. list of all compounds screened in this study and their stock concentration.


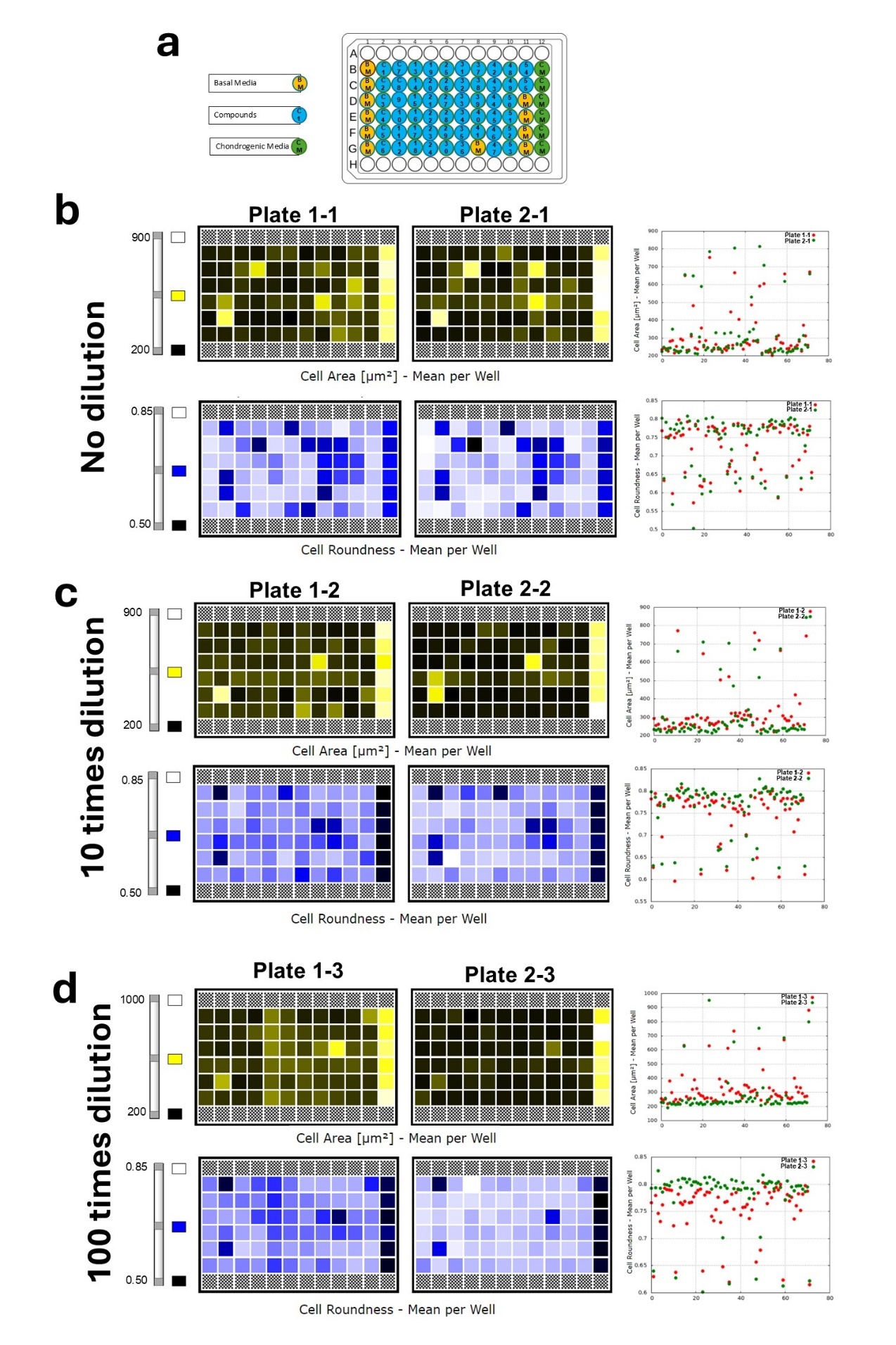


**Figure S4.** (a) Schematic design of the 96-well plate layout, including sample placements. (b,c,d) Effects of all bioactive compounds on cell morphology including analysis of cell area and cell roundness for compounds at three concentration levels, yellow and blue wells indicate positive control and hit compounds (b) undiluted, (c) 10x dilution, (d) 100x dilution.

**a**

**
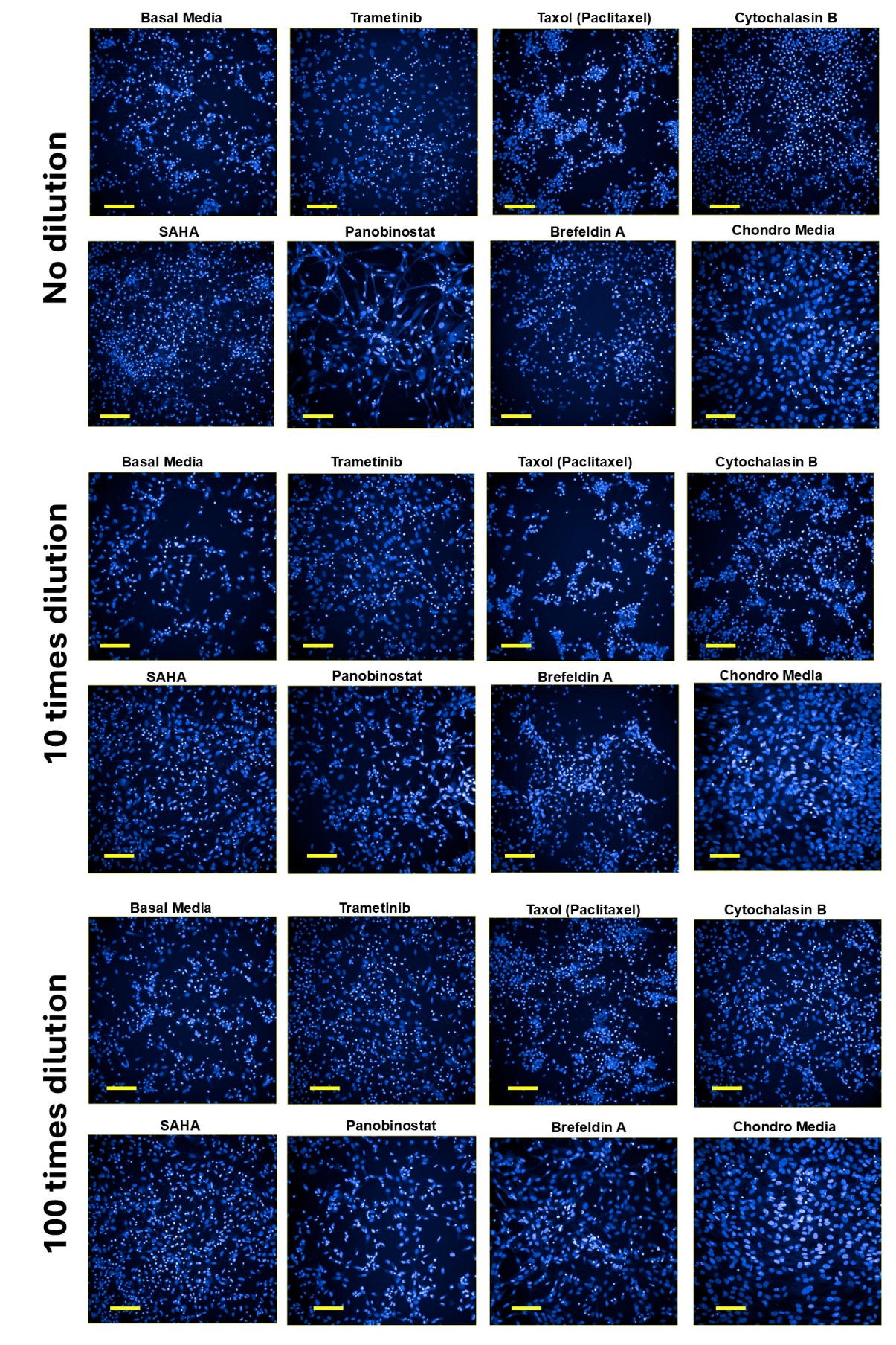
**

**c**

**b**

**Figure S5.** Representative fluorescence images of cells captured using the DAPI channel across 102 z-planes with 5 µm intervals. The images are shown as a projection of all z-planes, illustrating the total number of nuclei in samples treated with selected bioactive compounds at different concentration levels: (a) undiluted, (b) 10x dilution, and (c) 100x dilution. These projections highlight cell proliferation based on the total number of nuclei within the 3D structure under varying treatment conditions. Scale bars = 100 µm.


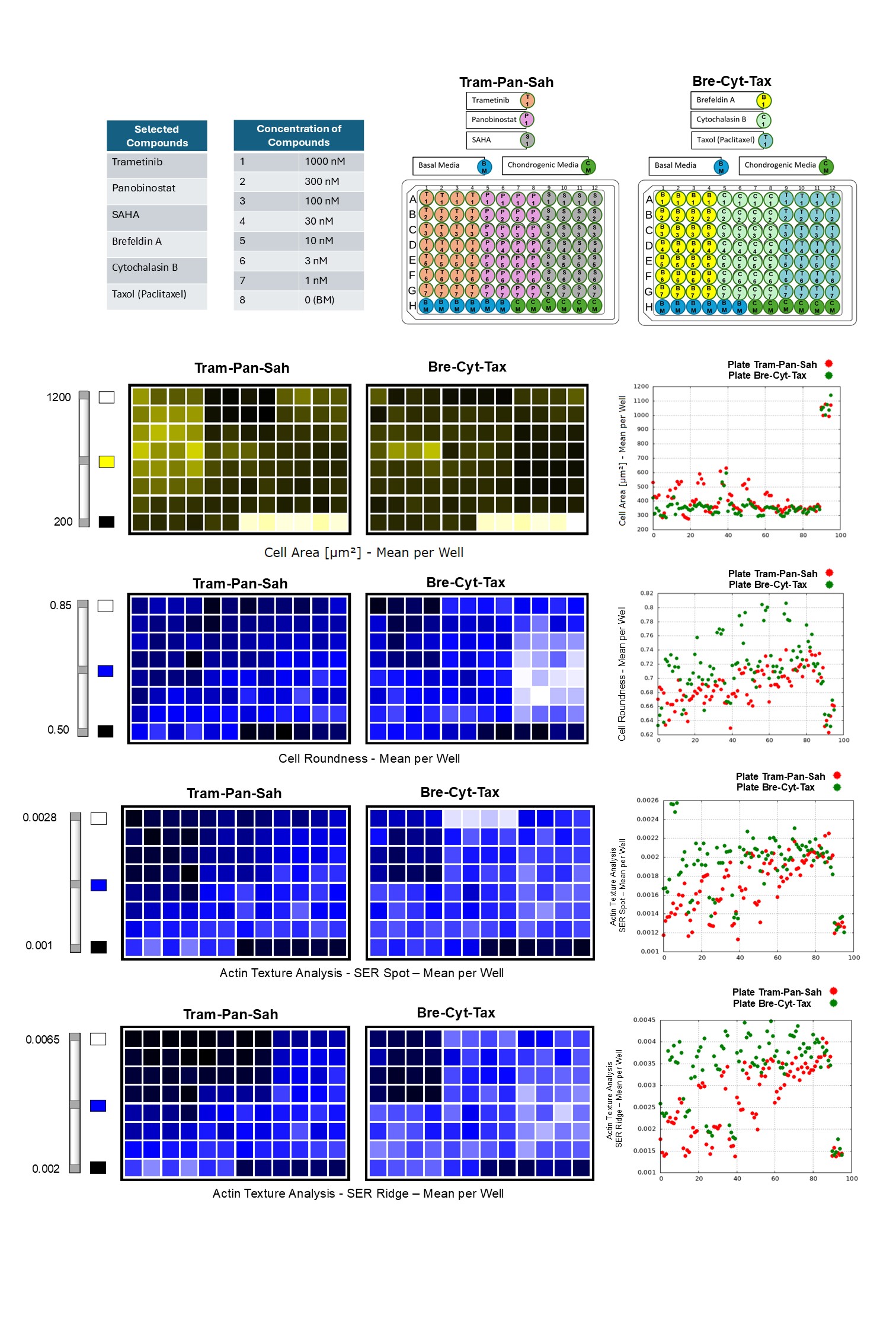


**f**

**e**

**d**

**c**

**b**

**a**

**Figure S6.** (a) List of selected compounds and the eight different concentrations screened in this experiment. (b) Schematic design of the 96-well plate layout, showing sample placements. (c-f) Effects of selected compounds and their dose responses on cell morphology and actin texture, including analysis of (c) cell area, (d) cell roundness, (e) actin texture analysis—SER Spot, and (f) actin texture analysis—SER Ridge, for selected compounds at eight concentration levels.


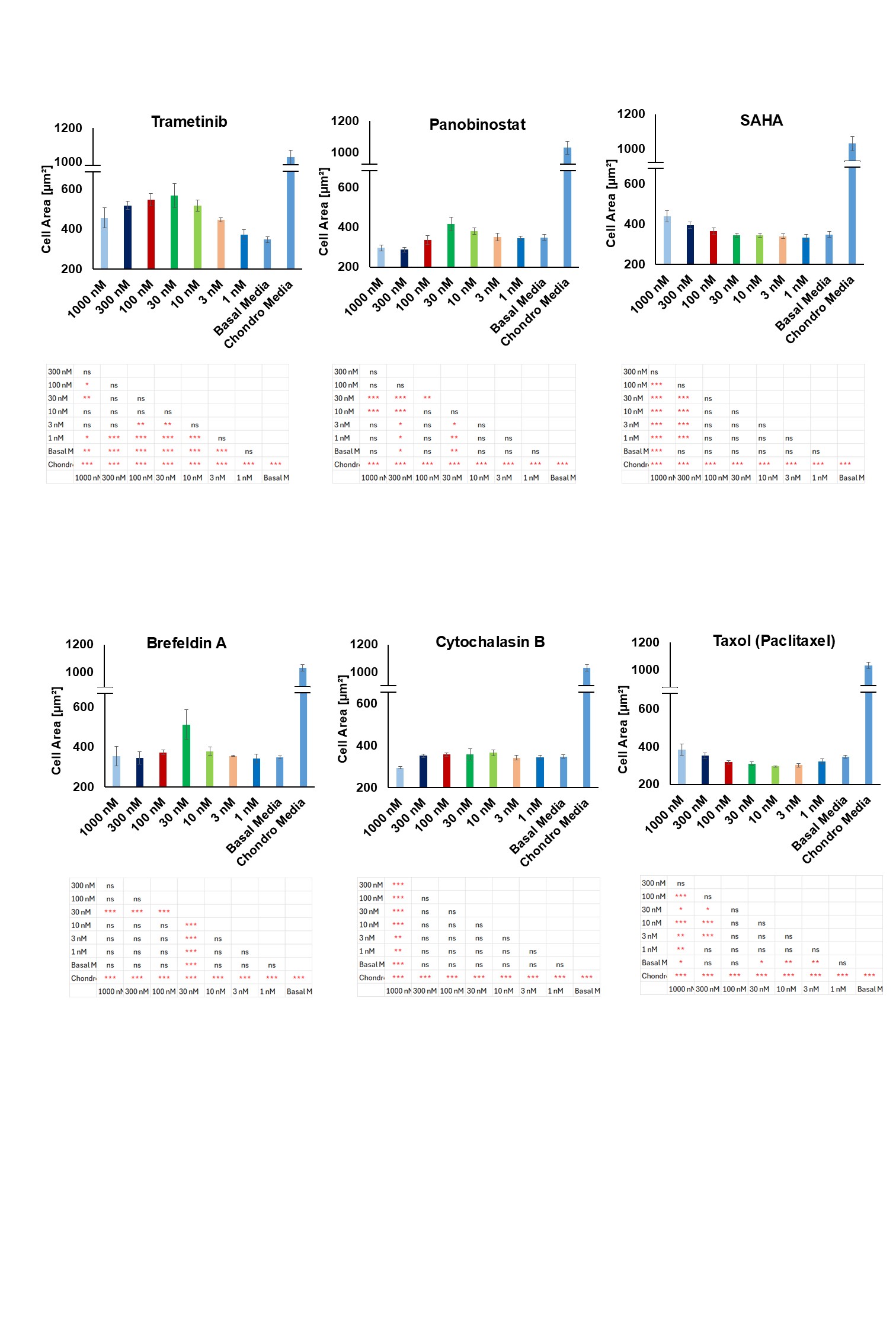


**Figure S7.** Effects of selected bioactive compounds and their dose responses on cell morphology with analysis of cell area at eight concentration levels (0, 1, 3, 10, 30, 100, 300, and 1000 nM). Data are expressed as mean ± SD, with statistical significance indicated by *p<0.05; **p<0.01; ***p<0.001; ns: not significant.


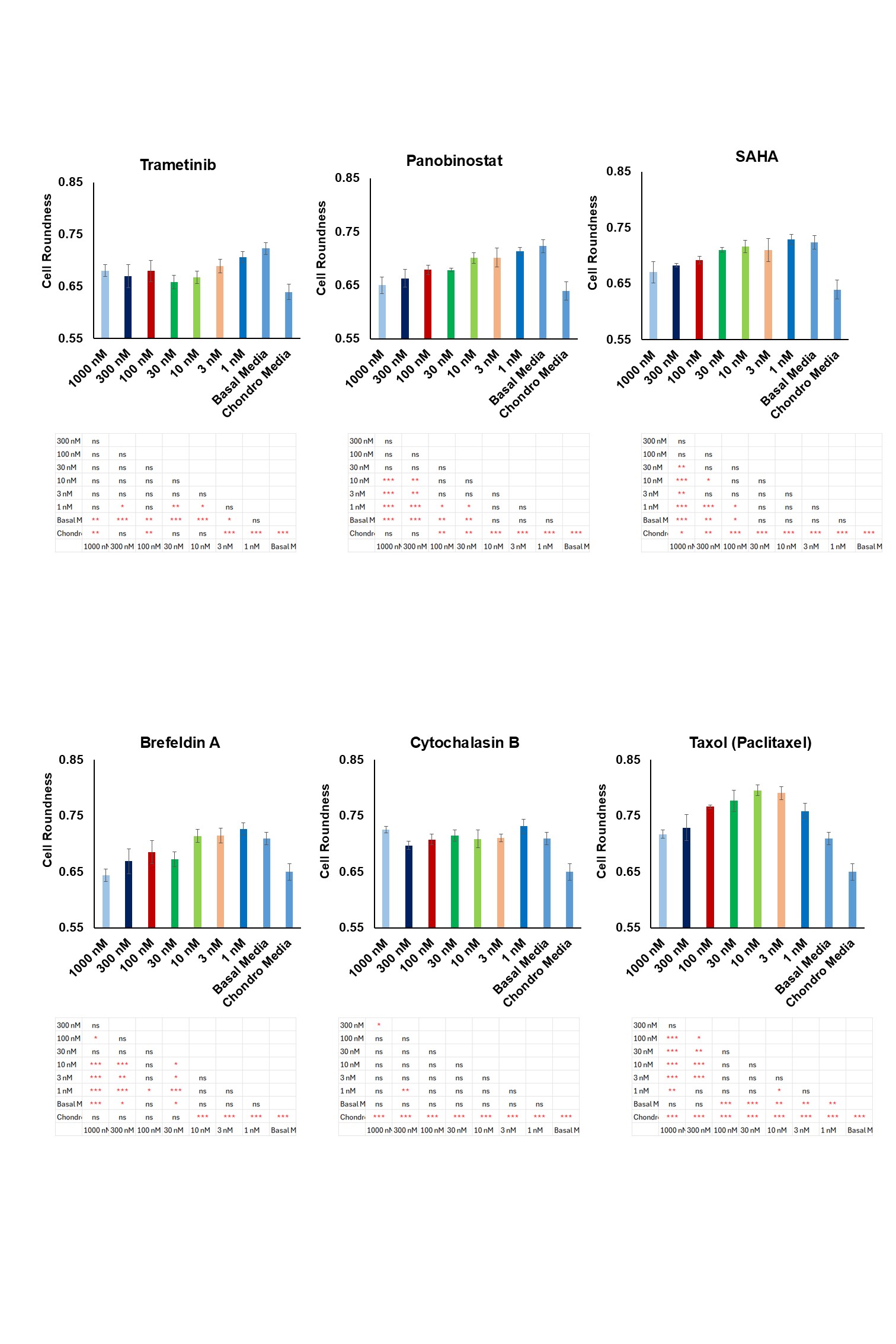


**Figure S8**. Effects of selected bioactive compounds and their dose responses on cell morphology with analysis of cell roundness at eight concentration levels (0, 1, 3, 10, 30, 100, 300, and 1000 nM). Data are expressed as mean ± SD, with statistical significance indicated by *p<0.05; **p<0.01; ***p<0.001; ns: not significant.


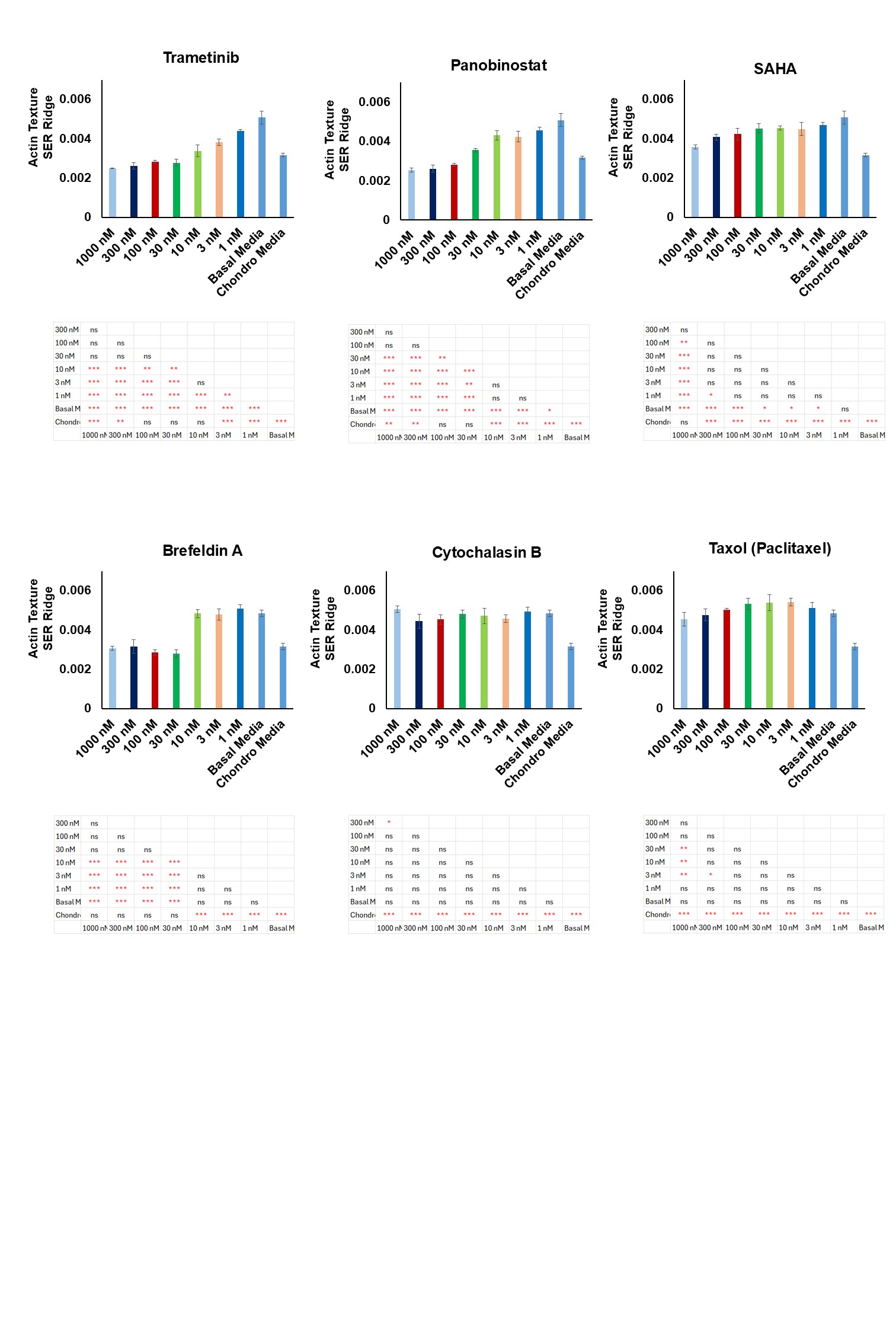


**Figure S9**. Effects of selected bioactive compounds on actin texture analysis through the SER Ridge indice at eight concentration levels (0, 1, 3, 10, 30, 100, 300, and 1000 nM). Data are expressed as mean ± SD, with statistical significance indicated by *p<0.05; **p<0.01; ***p<0.001; ns: not significant.


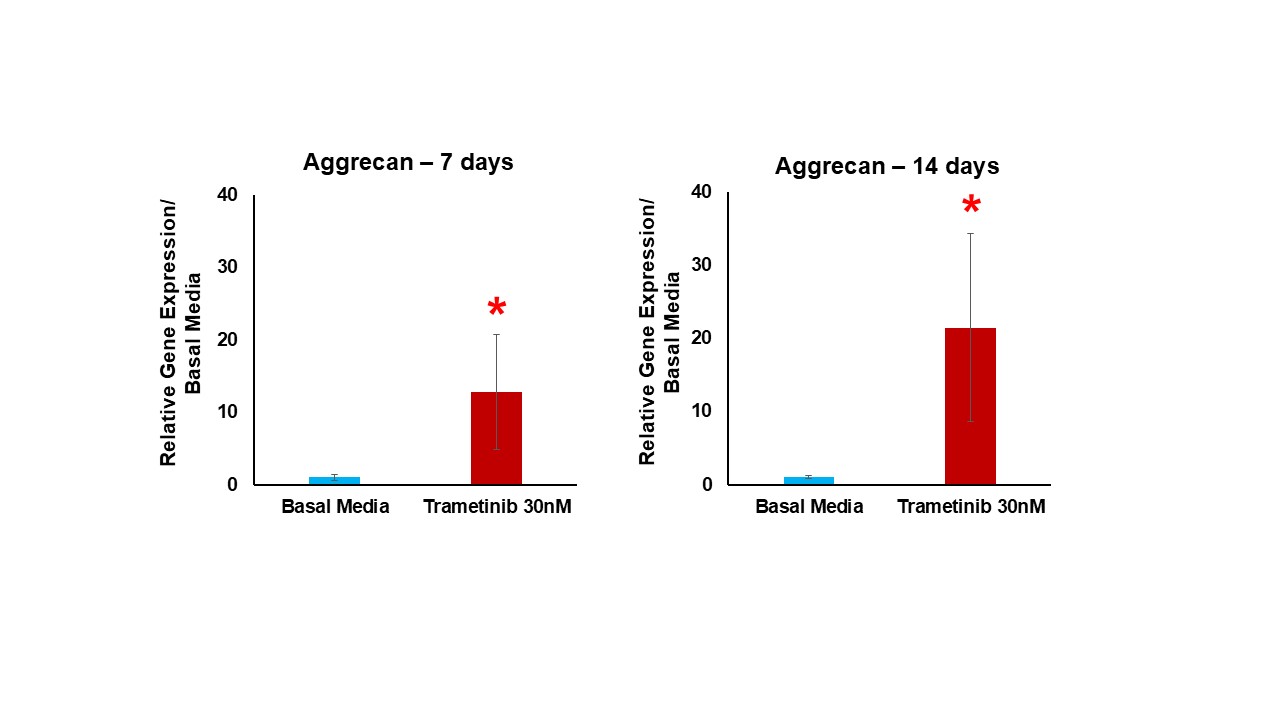


**a**

**b**

**Figure S10.** Trametinib (30 nM) significantly upregulated the expression of the ACAN (aggrecan) gene. (a) Gene expression analysis of Y201 cells treated with Trametinib for 7 days compared to the control (Basal Media). (b) Gene expression analysis of Y201 cells treated with Trametinib for 14 days compared to the control (Basal Media). Data are represented as mean ± SD, and statistically significant differences are marked with * for p < 0.05.

**Table S1.** Overview of selected bioactive compounds’ effects on chondrogenesis-related cellular parameters

| Compound | Morphology  (chondrogenic-like) | Migration | Proliferation | Actin Texture  (chondrogenic-like) |
| --- | --- | --- | --- | --- |
| Trametinib | ↑↑ | ↑ | ↔ | ↑↑ |
| Panobinostat | ↑ | ↔ | ↔ | ↑ |
| SAHA | ↑ | ↔ | ↔ | ↑ |
| Brefeldin A | ↑ | ↔ | ↔ | ↑ |
| Paclitaxel | ↔ | ↑↑ | ↔ | ↔ |
| Cytochalasin B | ↔ | ↑↑ | ↔ | ↔ |

↑↑: Strong effect; ↑: Moderate / partial effect; ↔: No Significant effect
